# Supplementary material for: How the COVID-19 Pandemic Affects Risk Awareness in Dentists: A Scoping Review
Source: Int J Environ Res Public Health. 2022 Apr 20;19(9):4971. doi: 10.3390/ijerph19094971 (PMC9103177; doi:10.3390/ijerph19094971)
Supplement: Supplementary file 1 [file ijerph-19-04971-s001.zip › ijerph-1654797/Table S1_Studies excluded after full-text review.pdf]

Table S1: Studies excluded after full-text review

Author	Reason of exclusion
Ahmed et al.	Data didn't meet inclusion criteria
Alkhalifah et al.	Data didn't meet inclusion criteria
Aly and Elchaghaby	Data didn't meet inclusion criteria
Anushya et al.	Data didn't meet inclusion criteria
Atas et al.	Data didn't meet inclusion criteria
Celik et al.	Data didn't meet inclusion criteria
Consolo et al.	Data didn't meet inclusion criteria
Haider et al.	Data didn't meet inclusion criteria
Javed et al.	Data didn't meet inclusion criteria
Kochhar et al.	Data didn't meet inclusion criteria
Kumar et al.	Data didn't meet inclusion criteria
Noushi et al.	Data didn't meet inclusion criteria
Novae et al.	Data didn't meet inclusion criteria
Pai et al.	Data didn't meet inclusion criteria
Plaza-Ruiz et al.	Data didn't meet inclusion criteria
Quadri et al.	Data didn't meet inclusion criteria
Reddy et al.	Data didn't meet inclusion criteria
Salgarello et al.	Data didn't meet inclusion criteria
Sarfaraz et al.	Data didn't meet inclusion criteria
Sarialioglu et al.	Data didn't meet inclusion criteria
Silvestre et al.	Data didn't meet inclusion criteria
Ugrappa et al.	Data didn't meet inclusion criteria
