# Supplementary material for: How the COVID-19 Pandemic Affects Risk Awareness in Dentists: A Scoping Review
Source: Int J Environ Res Public Health. 2022 Apr 20;19(9):4971. doi: 10.3390/ijerph19094971 (PMC9103177; doi:10.3390/ijerph19094971)
Supplement: Supplementary file 1 [file ijerph-19-04971-s001.zip › ijerph-1654797/Table S2_Studies excluded after data extraction.pdf]

Table S2: Studies excluded after data extraction

Author	Reason of exclusion
Ahmad et al.	Extracted data couldn't be included in the results
Aravind et al.	Extracted data couldn't be included in the results
Gambhir et al.	Extracted data couldn't be included in the results
Jafari et al.	Extracted data couldn't be included in the results
Jose et al.	Extracted data couldn't be included in the results
Jungo et al.	Extracted data couldn't be included in the results
Khanal et al.	Extracted data couldn't be included in the results
Martina et al.	Extracted data couldn't be included in the results
Maru et al.	Extracted data couldn't be included in the results
Sanchez-Perez et al.	Extracted data couldn't be included in the results
Shacham et al.	Extracted data couldn't be included in the results
Shubayr et al.	Extracted data couldn't be included in the results
Singh et al.	Extracted data couldn't be included in the results
